# Lived Experiences and Coping Strategies of Newly Recruited ICU Nurses in a Resource‐Constrained Hospital in Ghana

**DOI:** 10.1002/nop2.70950

**Published:** 2026-10-16

**Authors:** Kennedy Dodam Konlan, Angelina Koduah, Menford Owusu Ampomah

**Affiliations:** ^1^ Department of Adult Health, School of Nursing and Midwifery University of Ghana Legon Greater Accra Region Ghana

**Keywords:** coping, experiences, Ghana, intensive‐care units, mentorship, newly recruited nurses

## Abstract

**Aim:**

To explore the lived experiences and coping strategies of newly recruited intensive care unit (ICU) nurses in a resource‐constrained setting in Accra, Ghana.

**Design:**

An exploratory, descriptive qualitative study was conducted.

**Methods:**

We purposively recruited 12 newly recruited nurses from the ICUs of a teaching hospital in Accra. The participants were interviewed using a pilot‐tested interview guide. The interviews were audio‐taped, transcribed verbatim, and analysed using thematic analysis with the aid of Nvivo 11.

**Results/Findings:**

The main themes identified were experiences and coping strategies of the newly recruited nurses. On the sub‐themes under experiences, we identified the following: shift mismatches, unavailability of mentors and lack of supportive learning environment. On the coping strategies, the following sub‐themes were identified: self‐directed learning, the expectation of implementation of structured orientation programmes, regular evaluation and peer support networks.

**Conclusions:**

There is ineffective mentorship of newly recruited ICU nurses due to the absence of institutional mentorship protocols. Peer mentoring, periodic competence‐based assessment, and protected mentorship time are useful strategies that can be harnessed to promote nursing skills transfer in newly recruited nurses.

**Implications for the Nursing Profession:**

Nursing heads of hospitals should ensure structured mentorship protocols are put in place as well as protected mentor time to ensure effective nursing mentorship. Furthermore, heads of in‐service training units of hospitals need to institute competence‐based orientation programmes and periodic competence‐based assessment to help newly recruited nurses' transition effectively into the work environment.

**Impact:**

This study explored gaps in mentorship and identified strategies to promote effective nursing skills transfer. The findings will help nurse managers in hospitals ensure effective mentorship of newly recruited nurses.

**Reporting Method:**

We adhered to COREQ guidelines.

**Patient or Public Contribution:**

Twelve newly recruited nurses actively participated in the study.

**Abbreviation**
SSA: Sub‐Saharan Africa

## 1 Introduction

The transition from university or college to practice in clinical settings can be difficult, stressful and emotionally draining for newly recruited nurses (Bergs et al. 2022). There are concerns about the ability of newly recruited nurses to cope with and provide safe nursing care, particularly those working in sub‐Saharan Africa (SSA) where the health care system is extremely under‐resourced (Konlan et al. 2022). Newly recruited nurses from SSA therefore face a significant amount of stress when they initially enter clinical settings, especially in intensive care units (ICUs) because they lack the confidence and are exposed to unexpected situations (Aldosari et al. 2021; Andrén and Hammami 2011; Koduah et al., 2026). Studies (Bergs et al. 2022; Gardiner and Sheen 2016; Woo and Newman 2019) elsewhere suggest that the first year of practice as newly recruited nurses is regarded to be stressful and associated with reality shock, burnout and other negative experiences. The process of transitioning from being a nursing student to professional practice in a resource‐constrained setting is thus an arduous and possibly frustrating experience for most newly recruited nurses (Zaitoun et al. 2023), so social support in the workplace is crucial if newly recruited nurses are to have good transitional experiences (Bergs et al. 2022; Mtegha et al. 2022; Koduah et al., 2026; Smith and Hill 2019).

Confusion, uncertainty and stress are frequently linked to transitioning into the job of a practicing nurse and overseeing patients' lives and health (Serafin et al. 2022). Task‐oriented ambiguity, inadequate coaching and preceptor skills, a lack of patient care abilities and knowledge and difficulty accessing work‐related logistics have all been identified as the main difficulties newly recruited nurses' encounter (Atakro et al. 2019; Bergs et al. 2022; Mtegha et al. 2022; Smith and Hill 2019). For nurses transitioning from general clinical practice into critical care nursing as a specialty, the responsibility on their shoulders goes beyond the provision of comprehensive nursing care to patients (Innes and Calleja 2018; Zaitoun et al. 2023) but they are supposed to ensure patient safety, advocate for quality care and contribute to a supportive work environment (Gautam et al. 2024). As they strive to provide safe nursing care despite heavy workloads and increased accountability and responsibility for their patient care, this transition can be particularly difficult for these nurses (Gularte‐Rinaldo et al., 2010). As Irandoost et al. (2022) showed, the transition from school or general practice into a critical care nurse role, which comes with more challenging and emotionally burdensome tasks, is often accompanied by feelings of perplexity, anxiety and stress.

Newly recruited nurses are novice and often enter the workplace with the unrealistic expectation that they will be able to “hit the ground running” despite working in resource‐constrained environments (Egerod et al. 2021; Gularte‐Rinaldo et al. 2023; Lalithabai et al. 2021). However, these settings, especially those in Ghana, frequently have severe workloads and poor nurse–patient ratios (Konlan et al. 2022), which may hinder adequate support for newly recruited nurses as they transition from student to professional roles, complicating their adjustment and integration into clinical practice. Consequently, there is a pressing need to understand the lived experiences of newly recruited nurses and how these newly recruited nurses cope with the new work environment. Gaining insight into their specific challenges can inform strategies to better support newly recruited nurses to integrate better into the work environment and improve the quality of care rendered to patients, especially when a lot of experienced nurses are being lost to the developed worlds due to the rapid exodus of health workers from SSA and other developing countries.

### 1.1 Aim

We explored the lived experiences and coping strategies of newly recruited intensive care unit nurses in a resource‐constrained setting in Accra, Ghana.

### 1.2 Theoretical Framework Underpinning the Study

This study adapted the organizational socialization model to underpin it. The model has five (5) main constructs, namely new employee characteristics, newcomer tactics, organizational effort, adjustment measures, and outcomes but this study adopted only four of the constructs (Dirks and de Jong 2021; Gularte‐Rinaldo et al. 2023; Svensson et al. 2024). We excluded employee characteristics such as personality traits, motivation and prior experience. According to the model, feedback‐seeking behaviour, for instance, allows new nurses to adjust more rapidly and learn from their mentors' expertise. Also, newcomer tactics play a significant role in developing trust and rapport between mentors and newly recruited nurses, fostering open communication. The model also states that newcomer tactics are important because they are associated with several workforce outcomes. In general, socialization strategies are moderately associated with perceptions of fitness, job satisfaction, organizational commitment and intentions to quit. They are modestly associated with job performance and turnover (Dirks and de Jong 2021; Gularte‐Rinaldo et al. 2023; Svensson et al. 2024).

It is noted that when organizations provide clear and realistic information during recruitment, applicants are better able to self‐assess their fit with the job. Furthermore, newcomer adjustment has been associated with outcomes such as performance, job attitudes and retention (Dirks and de Jong 2021; Gularte‐Rinaldo et al. 2023; Svensson et al. 2024). The adjustment, which serves as the primary sign of the effectiveness of organizational socialization efforts, has been measured in two ways. The more common approach is to use independent measures of each primary adjustment indicator (role clarity, self‐efficacy and social acceptance; culture has not been typically included in this approach).

In the context of newly recruited nurses in ICUs, organization socialization model seeks to explain how newly recruited nurses learn the norms, values and expectations in the ICUs (Dirks and de Jong 2021; Gularte‐Rinaldo et al. 2023; Svensson et al. 2024). However, ineffective socialization can lead to turnover and decreased job satisfaction (Dirks and de Jong 2021; Gularte‐Rinaldo et al. 2023; Svensson et al. 2024).

Below provides a diagrammatic representation of the model underpinning the study (Figure 1).

**FIGURE 1:**
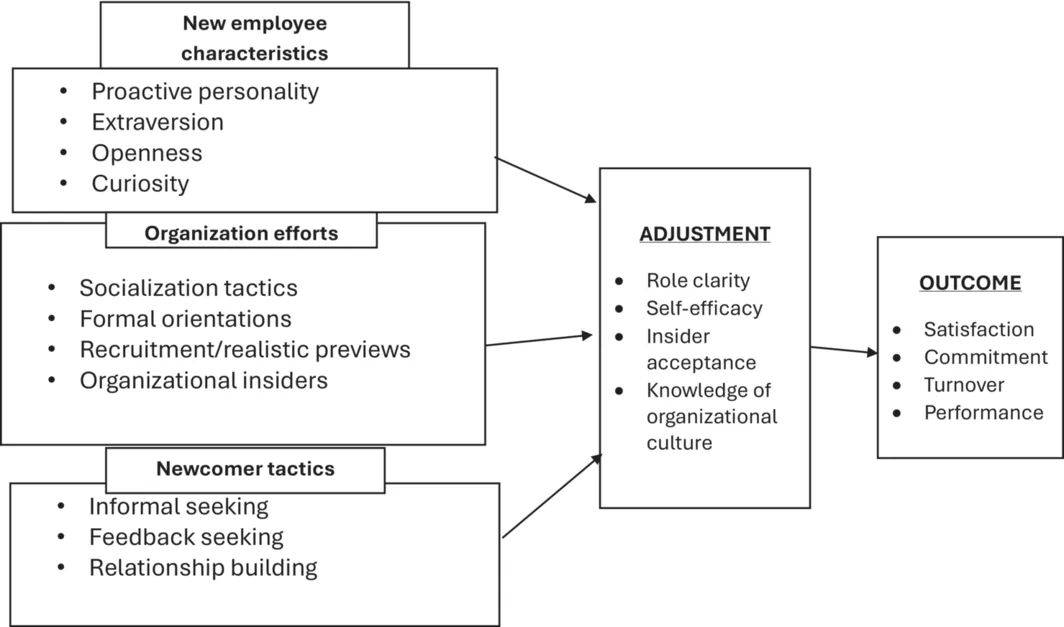
Organizational socialization model (Dirks and de Jong 2021).

## 2 Method

### 2.1 Study Design

We adopted an exploratory descriptive qualitative research approach in which in‐depth data was collected to explore and describe the lived experiences and coping strategies of newly recruited nurses providing care in intensive care units of a resource‐constrained setting in Accra, Ghana. This method was considered appropriate as it allowed the participants to vividly describe their perspectives on the topic under the study focusing on exploring the phenomena in detail and gaining a rich understanding of their individual perspectives (Clark et al. 2017; Creswell and Creswell 2017).

### 2.2 Study Setting

The study was conducted at the National Referral Hospital in Accra, Ghana. This Hospital is the third largest hospital in Africa and provides care for all categories of patients (Konlan et al. 2020). The hospital also provides sophisticated scientific treatment procedures in various subspecialties such as Neurosurgery, Paediatric surgery, Dental/Oral maxillofacial, Ophthalmology, Ear, Nose & Throat (ENT), Renal, Orthopaedics, Oncology, Dermatology, Reconstructive Plastic Surgery, Cardiothoracic Surgery and Radiotherapy & Nuclear Medicine. The hospital was selected because it is the largest hospital in Ghana with myriad of intensive care units with considerable number of newly recruited nurses due to the rising turnover of experienced staff (Koduah et al., 2026; Konlan et al. 2020), making it suitable to draw the study participants. The participants for the study were from the main ICUs of the study setting comprising of Medical ICU, Plastics and Reconstructive ICU, Cardiothoracic ICU and Neurological ICU. Each of these ICUs have an average bed capacity of Eight beds and provide twenty‐four‐hour nursing services. The hospital does not have an institutional‐specific mentorship policy with no specific protocols to guide mentorship even though mentorship is practiced but not in a structured form (Koduah et al., 2026).

### 2.3 Study Population

The study population was newly recruited nurses providing nursing care in the four intensive care units of the study setting. These were nurses who were new to the ICU environment at the study setting and had not previously worked in the said hospital but rather elsewhere as general nurses. The study setting had forty‐three of these newly recruited nurses as of March 2023.

### 2.4 Inclusion Criteria

The inclusion criteria were that only newly recruited nurses who had completed mandatory internship programme as required per their training and had worked within six to twelve months post completion of mandatory internship programme at the four main ICUs of the study setting. Also, only newly recruited nurses who were at post as of March 2023 were included in the study.

### 2.5 Sampling Technique and Sample Size

We adopted a purposive sampling technique to recruit the participants, and the sample size was determined at data saturation which was achieved at the completion of the twelfth (12) interview (Koduah et al., 2026). Specifically, we observed data redundancy upon completion of the tenth interview, and we observed that no new meanings were being elicited after the twelfth interview; hence meaning saturation.

### 2.6 Selection of Participants and Data Collection

Data collection involved in‐depth interviews in English lasting about 30–45 min which took place in the months of March to May 2023 in the nurses' restrooms of the various ICUs at the study setting. This was after ethical approval was granted by the Institutional Review Board of the study setting.

Recruitment of participants was done at the four intensive care units of the study setting on weekdays by the authors. We obtained a list of eligible participants from the nursing administration in charge of the four units. We then gave each eligible participant the information sheets after meeting them at the rest rooms of the four ICUs. We carefully and thoroughly explained the purpose of the study to those participants before obtaining written informed consent.

A pilot tested semi‐structured interview guide (Data S1) was developed and pilot‐tested among similar participants at another teaching hospital located in the Ashanti Region of Ghana, and it had two parts: A and B. Part A collected background information on participants such as age, duration of work, and nursing qualifications. Part B of the interview guide had open‐ended questions which provided an opportunity for the authors and the participants to delve deep into the subject area being discussed.

The in‐depth interviews which were carried out in English were audio‐recorded and transcribed verbatim. Every participant was given the opportunity to read and fill out the information sheet before each interview.

Throughout the course of gathering data, the researchers maintained a field journal. The diary entries were organized in chronological order based on the time and date of each interview. Participants' non‐verbal clues, such as facial expressions, were recorded in the field diary. Additionally, we documented any other events that occurred during data collection that influenced the collected information.

### 2.7 Data Analysis

The data analysis was done concurrently with data collection with the aid of Nvivo 11. We adopted the thematic analysis approach to data analysis as recommended in literature (Braun et al. 2014). The interviews that were audio recorded were transcribed word for word as soon as they were concluded. The audio transcripts in English were confirmed as verbatim transcripts of the audio files. The verbatim transcripts and the field notes recorded in the diary were used in the thematic analysis. The reflexive thematic analysis which was done concurrently with data collection involved six steps: familiarization with the data, generating codes, generating themes, reviewing themes, defining and naming themes and producing the report. The 1st and 3rd authors transcribed the audio files verbatim and immersed themselves in the data through repeated reading and listening. The data were systematically coded and sorted codes put into potential themes using mind maps and thematic tables. Specifically, we created initial codes that captured significant portions of information within the content of the transcripts from the interviews. The 1st and 3rd authors independently did the coding of the transcribed data and thereafter the two of them met and resolved any coding discrepancies before proceeding with the analysis. We then diligently looked for patterns, connections and recurring ideas. These recurring patterns and ideas provided the foundation for identifying the themes. These themes were carefully reviewed and scrutinized to see whether the themes accurately and comprehensively reflect the nuances and depth of the data. Each validated theme was then precisely defined and given a descriptive name. These well‐defined themes were eventually used to write the results section. We ensured that throughout this process, reflexive journaling and regular discussions among the three authors were conducted to ensure the rigour and credibility of the analysis.

### 2.8 Data Quality Control

To ensure quality control in the data collection, the interview guide was pilot tested at a teaching hospital in the Ashanti Region of Ghana among a similar population. This exercise helped to ascertain the clarity of the questions in the interview guide and determined whether the questions sought to answer the research questions. Questions that produced narrow and restrictive responses were modified to include the words “describe” or narrate to broaden the responses and to give the participants the opportunity to speak more on the topic. During the data collection, the researchers adhered strictly to methodological rigour as described below and also enhanced member checking with the participants to ensure that their views had been properly represented in the transcripts from the interviews. At the end of the data collection, the audio transcripts in English were confirmed as verbatim transcripts of the audio files so as to ensure the quality of the data collected was maintained.

### 2.9 Trustworthiness

To ensure credibility, the interview guide (Data S1) was pilot tested among two participants who met the inclusion criteria at a similar setting in Kumasi. This allowed the researchers to make necessary modifications to aspects of the interview guide that seemed not to elicit responses that were relevant to the objectives of the study. Additionally, a purposive sampling technique was employed to ensure only newly recruited nurses who could describe the characteristics of mentors and newly recruited nurses that could promote effective mentorship were engaged. Probing and iterative questioning were also used to elicit responses from participants and situations where there were ambiguities in the responses; clarifications were sought from the participants. To achieve transferability, the research process was described in detail so that others can evaluate the applicability of data to other contexts and settings. Records of the transcribed interviews and the analysis, as well as the results of the study, were kept for audit trail and are in the custody of the 1st author. To ensure dependability in the study, a semi‐structured interview guide was used for all the interviews to ensure consistency in the line of questioning among the participants. Again, a detailed description of the study design, sampling method, data collection and analysis was also documented. To ensure confirmability, the context of data collection was documented in a field notebook during the interviews. This enhanced the interpretation of the data during analysis to reflect the exact responses of the participants. The authors also bracketed their experiences and presuppositions to avoid any influence in the interpretation and analysis of the data.

### 2.10 Reflexivity

The researchers who are professional nurses with precious work history of working in ICUs but now in academia tried to ensure that their personal biases did not affect the interpretation given to the data. However, there was no supervisory relationship between the researchers and the participants since the researchers had left the ICUs prior to the participants being recruited to the same. In a bid to ensure that power imbalances between the researchers and the participants did not affect the interviews, the researchers encouraged the participants to feel free to voice out their concerns on the research topic without fear as the study was to help improve mentorship in nursing and had no impact on the participants personally. The researchers further assured the participants that the data would be kept confidentially and that no aspect of the data was accessible by their work supervisors, and this encouraged the participants to partake freely in the study. The researchers maintained an ethical and professional outlook throughout the study. They maintained their utmost fidelity to the advancement of science and gave a vivid account of the study findings without letting their personal biases and experiences influence the study findings.

## 3 Results

### 3.1 Socio‐Demographic Characteristics

The age of the participants ranged from 26 to 38 years. The length of work experience varied significantly, ranging from 7 months to 11 months. Regarding their nursing qualifications, the participants held either a Diploma in Nursing or a Bachelor of Science (BSc) in Nursing, with some holding both.

Table 1 gives a summary of the socio‐demographic characteristics of the participants.

**TABLE 1:** Socio‐demographic data of the study participants.

Participant	Age (in yrs.)	Length of work	Nursing qualification
P1	30	7 months	Diploma & BSc in Nursing
P2	36	7 months	BSc in Nursing
P3	30	11 months	Diploma in Nursing
P4	27	11 months	BSc in Nursing
P5	27	11 months	Diploma in Nursing
P6	38	10 months	BSc in Nursing
P7	26	11 months	BSc in Nursing
P8	30	7 months	BSc in Nursing
P9	30	9 months	Diploma in Nursing
P10	30	10 months	Diploma in Nursing
P11	33	7 months	Diploma in Nursing
P12	29	8 months	Diploma in Nursing

The themes and sub‐themes that were generated from the data are shown in Table 2.

**TABLE 2:** Themes and subthemes generated from the data.

Theme	Subtheme
Experiences	Shift mismatches in the pairing of newly recruited nurses with mentors Unavailability of mentors with field‐specific expertise Lack of Supportive and collaborative learning environment
Coping strategies	Self‐directed learning by newly recruited staff Expectation of effective structured orientation and mentorship programme Regular evaluation and feedback Peer support networks

### 3.2 Experience of Newly Recruited Nurses

The study uncovered mismatches in the shifts assigned to newly recruited nurses and that of the senior nurses allocated to them as mentors, with some of them being assigned general nurses as mentors instead of senior critical care nurses. The participants further claimed the learning environment was unsupportive of new nurses. The details of the sub‐themes are described below:

#### 3.2.1 Shift Mismatches in the Pairing of Newly Recruited Nurses With Mentors

A major finding across participants' interview transcripts was the challenge posed by shift mismatches, which led to reduced interaction time between mentors and newly recruited nurses. This mismatch affected the quality of learning, with newly recruited nurses often having to rely on self‐learning due to the absence of mentors during critical periods.

Initially when I went there, we were running the same shift…As time went on, we were not doing the same shift, so it was like the person that was supposed to teach me was not always available…(P 01).

The challenges I will say I encountered were that most times, they placed me and the first mentor I had on different shifts. So, it became difficult to learn certain procedures from her due to the shift mis‐match. (P 05)

There are times I am for morning shift and my mentor will be on the night shift so how do we even meet for her to teach me what I need to know. Sometimes the mentors will go on leave, and you find yourself stuck, the only people you can talk to are your senior colleagues, some of them are willing to teach, others are not so willing. They don't have time for you at all. (P 09)

The major challenge I encountered was the timing. You may be on the morning shift and your mentor in a different shift, so you don't get enough time to interact. (P 10)

The lack of consistent pairing on the same shifts created gaps in knowledge transfer and limited the participants' ability to learn directly from mentors.

Me personally, my mentor and I do not normally meet. We meet once a while. I do more of the morning shifts, she does more of the afternoons. So, there was enough time for her to teach me one or two things. (P 01)

In some cases, the participants had to depend on online resources or colleagues for guidance, potentially affecting their confidence and practical skills in critical care.

So sometimes I have to rely on the internet for certain information if I am not sure of what I am doing but my boss or mentor is not around. (P 06)

I ask colleagues and also make use of google sometimes if the senior nurse I am under has not come that day. (P 11)

#### 3.2.2 Unavailability of Mentors With Field‐Specific Expertise

Another challenge centred around the pairing of newly recruited nurses with mentors who lacked specific expertise aligned with the newly recruited nurses' goals. Some participants expressed a desire for mentoring from critical care nurses rather than general nurses to gain specialized skills and knowledge in critical care. Some of the participants' quotes were:

I felt like I should be given to or I should have been paired with a critical care nurse…rather than a normal general nursing officer who doesn't have so much background of critical care nursing. (P01)

One key challenge I experienced was the unfair pairing…goals and targets set were not achieved. (P08)

Some participants were of the view that not being paired with critical care nurses affected the experience they were supposed to have in the critical care setting

If you are under a senior nurse who is also in the field, you know that you are getting the right experience and maybe leaning the right things. (P 09)

It can be very frustrating when you are in a new environment, and you don't know what to do and there is no one to teach you what you should know. (P6)

#### 3.2.3 Lack of Supportive and Collaborative Learning Environment

The participants claimed that there was a lack of supportive and collaborative learning environment. Below are some quotes from the participants.

I would say I experienced many challenges because my mentors were always unavailable and unwilling to help and I had no reasons for their conduct…. (P04)

…Some too are very unwilling to help and even when you call for help…. (P08)

My mentor was supposed to teach me virtually everything in the ICU but the least I say about it the better, he did not care at all. (P07)

The mentor gets to provide corrective feedback whenever you do something and also the mentor gets to evaluate whatever you have done but this does not exist here. (P12)

You come to work and there is no one to help you, it makes you stranded. (P03)

There is lack of support and collaboration among the mentors/professionals, everyone is just inward and does not care about us. (P11)

### 3.3 Coping Strategies for Newly Recruited Nurses

The measures adopted by the participants to help them transition into the new work environment included self‐directed learning by newly recruited nurses, structured mentorship and orientation programmes, regular evaluations and feedback, and building of peer networks. The specific sub‐themes are discussed below.

#### 3.3.1 Self‐Directed Learning by Newly Recruited Nurses

While shift mismatches were a barrier, the participants showed resilience by seeking alternative ways to learn. Although there were challenges and stressful situations, this experience fostered self‐reliance which ultimately helped participants develop problem‐solving skills and autonomy in their practice. Some of the participants stated:

Sometimes it's fun, it is challenging because you don't know what exactly to do but as you go on, you have to do it anyways and have to do it on your own. (P02)

I do it and then my mentor comes and I tell her I have done it… Sometimes it's fun, it is challenging. (P06)

#### 3.3.2 Expectation of Effective Structured Orientation and Mentorship Programme

The participants stated that their expectation of effective structured orientation and mentorship programmes helped newly recruited nurses to cope effectively with the new work environment. Below are some quotes from the participants:

The hospital needs to ensure we have a structured orientation and then they introduce mentorship during the orientation and throughout the work environment. (P11)

The mentors must be assigned to newly recruited nurses during orientation to help us adjust to the work. We need dedicated mentor‐mentee time to help us cope (P09)

The facility should draw up a good orientation program so that the mentors won't just bombard us with things at a go. (P03)

Things should be taken one step at a time, we can have well designed orientation, followed by introduction and pairing of mentors to newly recruited nurses. (P04)

This, they (the participants) believe, would smoothen their transition into their roles and meet mentorship expectations effectively. Some of the participants stated below:

If the orientation is step‐by‐step with a structured mentorship, we will be able to learn and get hands‐on experience in the intensive care units. (P08)

The only mentorship program that was available to us was orientation which lasted for like 1 month to 4 months and was basically based on nurse demonstration and completion of competencies. (P3)

#### 3.3.3 Regular Evaluations and Feedback

The study identified regular evaluation and feedback of the progress of newly recruited nurses as one adjustment measure that the facility could employ to help newly recruited nurses cope with the demands of work. Some of the participants' quotes were:

The role of the employer is evaluating the mentorship program. The employer needs to evaluate each session of mentorship to know the strengths and weaknesses of the new nurse. (P05)

Yes, I will say once in a while the In‐Charges do assessments to see how well we are doing; we those who just came new. (P 09)

I think well‐meaning feedback assist us to adjust to the work environment. (P 01)

Such regular evaluations helped in assessing the progress of newly recruited nurses and identifying areas for improvement. Some of the participants' quotes included:

…if they do regular evaluations, they the mentors can see the progress we are making. (P10)

Regular evaluation and feedback is what I encourage so I know where I am doing well and areas that I need to improve. (P 03)

#### 3.3.4 Peer Networks

Establishing peer networks among newly recruited and existing nursing staff fosters a collaborative learning environment and provides additional support mechanisms. Some of the participants' quotes were:

It's not necessarily your mentor that can only help you. You can seek advice from your senior colleagues as well or even your peers. My mates have helped me adjust to the work demands better than even my mentor. (P11)

I sometimes read online and if I have challenges, I consult my mentor if she is around and if she is not, I consult other colleagues on the ward or my mates, and this has helped me. When we share ideas, we learn a lot among ourselves and that has indeed helped us. (P5)

Sometimes you want the new staff to be seen as peers by the old staff while these old staff help we the new staff overcome the challenges of work. (P 01)

I sometimes feel isolated especially when I am duty with only old staff, It gets better when the old staff trat you like a peer and wish to support me cope with the work. (P 04)

Peer mentoring was reported by the participants as one effective way that helped them cope with the demands of the work.

## 4 Discussion

Transitioning from formal training into a high‐pressure clinical environment such as critical care unit is challenging for newly recruited nurses. This finding is similar to those found in other studies (Aldosari et al. 2021; Mtegha et al. 2022; Wang et al. 2022). This initial period of adjustment is marked by high expectations, self‐doubt and a need for substantial mentorship to bridge the gap between training and practice (Dirks and de Jong 2021; Gularte‐Rinaldo et al. 2023; Svensson et al. 2024). In the present study, newly recruited nurses had challenges with mismatched shifts with their mentors as they worked morning shifts while mentors were assigned different shifts. This limited opportunities for direct interaction and hands‐on learning and possibly reduced available time for guided practice and feedback. This led some newly recruited nurses to rely on self‐learning, online resources or assistance from other colleagues, which sometimes left them feeling unsupported. This finding corroborates what Burry et al. (2020) reported in their study which they showed that irregular scheduling and shift mismatches can disrupt the continuity of mentoring relationships and limit newly recruited nurses' ability to learn through observation and guided practice. This finding also reinforces the notion that effective mentoring requires consistent availability and time for interaction, as noted in studies emphasizing the importance of structured pairing of schedules for newly recruited nurses (Choi et al. 2016; Egerod et al. 2021; Gautam et al. 2024; Irandoost et al. 2022).

In addition to mismatch in shift scheduling with newly recruited nurses, the study found that some of the newly recruited nurses were paired with general nurses rather than senior critical care nurses, which they felt hindered their specialized learning. The lack of alignment between newly recruited nurses' needs and mentors' expertise created frustration and dissatisfaction, with newly recruited nurses expressing a preference for mentorship from professionals with critical care experience. Similar to findings from the current study, previous research highlights the negative impact of this misalignment on newly recruited nurses' confidence and skill development in specialized fields like critical care nursing (Woo and Newman 2019). According to Hill et al. (2022), mentorship effectiveness is shown to be enhanced when mentors possess relevant expertise, allowing them to provide context‐specific guidance and feedback, which is particularly crucial in high‐stakes environments like critical care units (Hill et al. 2022). Thus, the situation as pertaining to the participants in this study is concerning and can affect the success of the entire mentorship and orientation programme.

The participants from this study claimed that they developed resilience by actively seeking other ways to learn. They used online resources, consulted colleagues and adopted a more independent approach. The self‐directed learning approach adopted mirrors findings in studies where newly recruited nurses took greater initiative in their learning due to mentorship gaps (Egerod et al. 2021; Woo and Newman 2019). Such adaptive strategies have been shown to be useful in the transition period as it helps the newly employed nurse to build resilience, autonomy and confidence, especially in situations with limited mentor support (Dirks and de Jong 2021). Although self‐directed learning can enhance problem‐solving skills (Aldosari et al. 2021; Atakro et al. 2019; Bergs et al. 2022), it is recommended that it complements rather than substitutes direct mentorship in critical care settings (Flinkman and Salanterä 2015; Gardiner and Sheen 2016; Smith and Hill 2019).

A few of the newly recruited nurses reported positive mentorship experiences, where mentors were consistently available, supportive and actively involved in teaching. These mentors provided constructive feedback and practical training, helping newly recruited nurses build confidence, competence and motivation. Positive interactions helped the newly recruited nurses feel more equipped to manage critical care tasks, enhancing their transition into the role (Flinkman and Salanterä 2015; Gardiner and Sheen 2016; Smith and Hill 2019). Positive mentorship experiences, where mentors are supportive and provide hands‐on training, have been shown to drive successful mentor‐mentee relationships with improved mentee performance and satisfaction (Bergs et al. 2022). To that end, supportive interactions between newly recruited nurses and mentors as well as among newly recruited nurses are crucial for newly recruited nurses' adaptation to high‐pressure environments, as it encourages skill development and emotional support (Atakro et al. 2019; Burry et al. 2020; Flinkman and Salanterä 2015; Gardiner and Sheen 2016; Smith and Hill 2019).

The expectation for a structured, step‐by‐step orientation programme aligns with previous studies showing that gradual, organized training reduces stress and supports competency development in new ICU nurses. Lalithabai et al. (2021) found that a well‐structured orientation inspires a positive attitude and helps new nursing staff demonstrate competence effectively (Lalithabai et al. 2021). However, unlike the ideal long‐term orientation described in Lalithabai's research, this study noted that most programmes lasted only one to four months, which participants found insufficient for comprehensive ICU skills mastery.

The emphasis on regular evaluations to assess newly recruited nurses' progress mirrors findings in prior studies (Bergs et al. 2022), which underscore how periodic assessments guide both newly recruited nurses and mentors in identifying strengths and areas needing improvement. Regular evaluations not only provide structured feedback but also allow for continuous professional growth, which is seen as essential in competency‐based training for ICU settings (Choi et al. 2016; Kilduff and Brass 2017; Svensson et al. 2024). Continuous mentorship beyond the initial orientation phase was highlighted in this study as critical for supporting newly recruited nurses. This finding aligns with studies indicating that ongoing mentorship is invaluable for skill reinforcement and confidence building. Continuous mentorship allows for error correction and learning opportunities, which are essential in high‐stakes environments like the ICU (Innes and Calleja 2018).

Lastly, the study identified peer networking as an adjustment measure that can enhance the mentorship/learning experiences of the nurses. Peer networks create supportive work environments in the ICU. It is an established fact that peer networks foster collaboration, shared learning and emotional support, helping new nurses integrate more comfortably into critical care roles (Bergs et al. 2022; Burry et al. 2020). Such networks also enable access to experienced colleagues for advice and support beyond formal mentorship channels (Aldosari et al. 2021).

This study adapted the Organizational Socialization Model to underpin it. The findings support the fact that newcomer tactics, organizational effort, and adjustment measures leading to better coping outcomes by newly recruited nurses as reported in literature and in line with the constructs of the organizational socialization model (Dirks and de Jong 2021; Gularte‐Rinaldo et al. 2023; Svensson et al. 2024).

### 4.1 Limitations of the Study

We excluded employee characteristics such as personality traits, motivation and prior experience which is one of the constructs of the organizational socialization model, and this should be the focus of future studies.

The study was restricted to a few nurses due to the design of the study, and this makes it difficult to generalize the findings of the study. Future studies can look at designs that will permit generalization.

The study findings could have been affected by social desirability bias and recall bias during the interviews even though we encouraged the participants to give a vivid account of their experiences.

The study was based on a single setting and cannot be generalized to other settings that were not studied.

The study used a cross‐sectional design making it difficult to infer causation, but the study provides some evidence about experiences of newly recruited nurses on mentorship as well as coping strategies adopted by the new nurses.

## 5 Conclusions and Recommendations

There is ineffective mentorship of newly recruited ICU nurses due to the absence of institutional mentorship protocols. Peer mentoring, periodic competence‐based assessment, and protected mentorship time are useful strategies that can be harnessed to promote nursing skills development and transfer in newly recruited ICU nurses in resource‐constrained settings.

It is recommended for nursing practice that nursing heads of ICUs of hospitals should ensure effective structured mentorship protocols are put in place as well as protected mentor time to ensure effective nursing mentorship and nursing skills transfer. Furthermore, heads of nursing in‐service training units of hospitals need to institute competence‐based orientation programmes and periodic competence‐based assessment to help newly recruited nurses' transition effectively into the work environment. Also, there is the need for nursing managers of ICUs to ensure effective planning of shifts between newly recruited nurses and experienced nurses to promote skill transfer from experienced nurses to newly recruited nurses.

## Author Contributions

**Kennedy Dodam Konlan:** conceptualization, investigation, methodology, validation, writing – original draft. **Angelina Koduah:** conceptualization, investigation, funding acquisition. **Menford Owusu Ampomah:** investigation, validation, methodology, supervision.

## Funding

The authors have nothing to report.

## Disclosure

We declare that we did not use artificial intelligence tools in preparing this manuscript.

## Ethics Statement

The study data was part of the protocol titled: “Transitional Experiences of Newly Recruited Critical Care Nurses on Mentorship: A Study at the Study Setting” which was approved by the Institutional Review Board of the study setting (REDACTED). Participants were made to sign a written informed consent to participate in the study after the objectives and purpose of the study were explained to them and were informed that participation is voluntary so only those who met the criteria and were willing to participate were involved in the study. Additionally, participants were assured of privacy, anonymity and confidentiality of information as well as their right to withdraw from the study at any point they wished to without any repercussions to their person.

## Consent

The authors have nothing to report.

## Conflicts of Interest

The authors declare no conflicts of interest.

## Supporting information


**Data S1:** Pilot tested interview guide.

## Data Availability

The data that supports the findings of this study are available in the supporting information (Data S1) of this article.
