# Supplementary material for: Lived Experiences and Coping Strategies of Newly Recruited ICU Nurses in a Resource‐Constrained Hospital in Ghana
Source: Nurs Open. 2026 Oct 16;13(10):e70950. doi: 10.1002/nop2.70950 (PMC13675602; doi:10.1002/nop2.70950)
Supplement: Supplementary file 1 [file NOP2-13-e70950-s001.docx]

**Supplementary file 1**

**Pilot tested interview guide**

Dear Study Participant,

This interview is designed to elicit information that will enable the researchers to describe **“Transition experiences of newly recruited nurses in the critical care setting”.** The purpose of this face-to-face interview is to obtain structured feedback to achieve the objectives of the study.

**SECTION A – PARTICIPANT’S PROFILE**

Please tell me about yourself as much as you can remember

1. What is your age?
2. How long have you been a nurse in this unit (Intensive Care Unit)?
3. What is your Nursing education qualification as at now?
4. How many years have you been in this current hospital?
5. How many years of experience have you worked as a critical care nurse?
6. Do you work full time, part time or casual in the intensive care unit?
7. What type of Intensive Care Unit do you mostly work in this hospital?

**SECTION B:**

8. Explain your current beliefs about mentorship?
9. Narrate the nature of mentorship that you experienced while working at the intensive care unit?
10. Describe the role that newly recruited critical care nurses play in mentorship programme?
11. Narrate the methods of communication that were used during mentorship in the intensive care unit?
12. Describe your perception of newly recruited critical care nurses about the role of employer in mentorship programmmes?
13. Narrate your experiences as a newly recruited critical care nurse in the ICU?
14. Narrate the barriers that you have encountered with mentorship? How have you overcome these barriers? How did you cope?
15. Narrate the impact of newly recruited critical care nurses’ characteristics on mentorship programmmes?
16. Did you gain any teaching skills from your mentor during your training and how would it help you in the future as a mentor?
17. How do you think mentorship programmmes might be better developed, implemented, and evaluated?
18. Are there any questions that you thought I might ask that I didn’t regarding your transition from school to work?

Thank you
